# The interplay between national and parental unemployment in relation to adolescent life satisfaction in 27 countries: analyses of repeated cross-sectional school surveys

**DOI:** 10.1186/s12889-019-7721-1

**Published:** 2019-11-28

**Authors:** Klara Johansson, Solveig Petersen, Björn Högberg, Gonneke W. J. M. Stevens, Bart De Clercq, Diana Frasquilho, Frank Elgar, Mattias Strandh

**Affiliations:** 1 Department of Global Health and Epidemiology, Umeå University, 901 87 Umeå, Sweden; 2 Department of Social Work, Umeå University, Umeå, Sweden; 3 Centre for Child and Adolescent Studies, Utrecht University, Utrecht, The Netherlands; 4 Department of Public Health, Faculty of Medicine and Health Sciences, Ghent University, Ghent, Belgium; 5 Chronic Diseases Research Center (CEDOC), NOVA Medical School, NOVA University of Lisbon, Lisbon, Portugal; 6 Institute for Health and Social Policy, McGill University, Montreal, Canada

**Keywords:** Adolescents, Life satisfaction, Unemployment, National factors, Health behaviour in school-aged children, HBSC

## Abstract

**Background:**

Previous research shows that parental unemployment is associated with low life satisfaction in adolescents. It is unclear whether this translates to an association between *national* unemployment and adolescent life satisfaction, and whether such a contextual association is entirely explained by parental unemployment, or if it changes as a function thereof. For adults, associations have been shown between unemployment and mental health, including that national unemployment can affect mental health and life satisfaction of both the employed and the unemployed, but to different degrees. The aim of this paper is to analyse how national unemployment levels are related to adolescent life satisfaction, across countries as well as over time within a country, and to what extent and in what ways such an association depends on whether the individual’s own parents are unemployed or not.

**Methods:**

Repeated cross-sectional data on adolescents’ (aged 11, 13 and 15 years, *n* = 386,402) life satisfaction and parental unemployment were collected in the Health Behaviour in School-aged Children (HBSC) survey, in 27 countries and 74 country-years, across 2001/02, 2005/06 and 2009/10 survey cycles. We linked this data to national harmonised unemployment rates provided by OECD and tested their associations using multilevel linear regression, including interaction terms between national and parental unemployment.

**Results:**

Higher national unemployment rates were related to lower adolescent life satisfaction, cross-sectionally between countries but not over time within countries. The verified association was significant for adolescents with and without unemployed parents, but stronger so in adolescents with unemployed fathers or both parents unemployed. Having an unemployed father, mother och both parents was in itself related to lower life satisfaction.

**Conclusion:**

Living in a country with higher national unemployment seems to be related to lower adolescent life satisfaction, whether parents are unemployed or not, although stronger among adolescents where the father or both parents are unemployed. However, variation in unemployment over the years did not show an association with adolescent life satisfaction.

## Background

Adolescents living with one or two unemployed parents report lower life satisfaction [1–4]. By extension, high *national* unemployment rates should imply lower national averages of life satisfaction among adolescents, because more adolescents would be expected to live with unemployed parents. This has however not been analysed before.

There is also a possibility that adolescents in general may be affected by high national unemployment rates, even when their parents are not unemployed [3, 5]. Among adults, national unemployment can relate negatively to mental health and life satisfaction among both the unemployed and the employed [6–8]. For young children, it has been hypothesized (and empirically tested) that macroeconomic crises only affect the child if the family is affected [9, 10]. Adolescents, however, are in transition between childhood and adulthood. They might be independently affected by high national unemployment, due to worries for their family or for their own future [11].

Although national unemployment may affect adolescent life satisfaction in general, the impact could be assumed to be *stronger* among those with unemployed parents. There is support for such cross-level interactions between national and personal unemployment in adulthood [6, 12], but research on this among adolescents is lacking.

Life satisfaction among adolescents is a relevant outcome to study in relation to national unemployment, in particular since life satisfaction has already been examined in relation to parental unemployment [1–4], and has been shown to correlate with other life circumstances and with mental health [13]. There are mixed trends in life satisfaction among adolescents in Europe between 2002 and 2010, with increasing life satisfaction in Estonia, Croatia, Lithuania, Latvia, Russia, Ukraine, Spain, Norway, Portugal and Belgium, and decreasing life satisfaction in Austria, Canada, Switzerland, Denmark, Finland, Greenland, Hungary and Macedonia [14]. Rising national level income has been found to be associated with rising adolescent life satisfaction in Europe and North America [15] but little is known about the importance of national unemployment for the adolescent life satisfaction. Among adults, earlier studies have analysed general well-being and self-reported general health both in relation to own unemployment [16–19] and national unemployment [6].

To fully understand the importance of national unemployment for individual life satisfaction, it is important to distinguish cross-sectional associations between countries as different from and not directly generalizable to temporal associations within countries [20]. We aim to disentangle cross-country associations from longitudinal associations on country level.

This study analyses how national unemployment levels are related to adolescent life satisfaction, across countries as well as over time within a country, and to what extent and in what ways such an association depends on whether the individual’s own parents are unemployed or not. This is done through combining national harmonized data on unemployment with individual data from repeated cross-sectional school surveys among adolescents aged 11, 13 and 15 years old in the years 2001/02, 2005/16 and 2009/10.

What this study contributes to previous knowledge is mainly to analyse the importance of national unemployment for the wellbeing of the age group of young adolescents, something that has previously been analysed mainly among persons who are of an age to be on the labour market. The role of parental unemployment in the above-mentioned relationship is an added contribution.

## Method

### Data

The Health Behaviour in School-aged Children (HBSC) study is a WHO collaborative cross-national study performed every fourth year in Europe and North America [21]. Each survey cycle involves a cross-sectional sample of adolescents aged 11, 13 and 15 years, recruited using two-stage random cluster sampling: first randomizing schools or school classes in each country, then inviting all students in the selected schools/classes. Approximately 1500 adolescents per age group and country are recruited, although many countries choose to have larger samples [22]. Questionnaires are administered in classrooms, and follow standardized instructions.

For the current study, open access data were used from the HBSC waves 2001/02, 2005/06 and 2009/10, for each country with at least 2 waves [23]. Scotland, Wales and England were combined as United Kingdom, and separate surveys in French and Flemish Belgium were combined as Belgium. Of 38 HBSC-countries that participated the relevant years, 27 countries had data on national unemployment as well as on the included survey variables for at least two cycles (Table 1), resulting in a sample of 386,402 respondents. Countries excluded due to lack of data were Armenia, Bulgaria, Croatia, Greenland, Israel, Lithuania, Macedonia, Romania, Russia, Switzerland and Ukraine.

**Table 1:** Descriptive statistics by country and year

		2002	2006	2010
All countries	National unemployment, %	7.63	7.36	9.41
	At least one parent unemployed, %	5.39	4.45	6.17
	Mean life satisfaction	7.55	7.57	7.59
	Sample size, n	107,786	142,024	136,592
Austria	National unemployment, %	4.27	5.64	5.33
	At least one parent unemployed, %	1.97	4.07	3.15
	Mean life satisfaction	7.95	7.83	7.58
	Sample size, n	4007	4662	4828
Belgium	National unemployment, %	6.99	8.52	8.20
	At least one parent unemployed, %	4.80	4.32	4.85
	Mean life satisfaction	7.72	7.70	7.59
	Sample size, n	6156	4132	7488
Canada	National unemployment, %	7.56	6.52	
	At least one parent unemployed, %	4.13	3.67	
	Mean life satisfaction	7.56	7.42	
	Sample size, n	4118	5591	
Czech Republic	National unemployment, %	7.73	7.69	7.38
	At least one parent unemployed, %	6.11	5.07	4.15
	Mean life satisfaction	7.45	7.28	7.51
	Sample size, n	4938	4671	4217
Denmark	National unemployment, %	4.53	4.36	6.87
	At least one parent unemployed, %	5.16	1.48	7.35
	Mean life satisfaction	7.72	7.82	7.53
	Sample size, n	3732	4621	3916
Estonia	National unemployment, %	11.80	6.98	16.40
	At least one parent unemployed, %	8.47	3.42	9.77
	Mean life satisfaction	7.17	7.64	7.67
	Sample size, n	3927	4408	4171
Finland	National unemployment, %	9.12	8.06	8.46
	At least one parent unemployed, %	6.33	5.54	6.11
	Mean life satisfaction	7.95	7.94	7.77
	Sample size, n	5222	4935	6465
France	National unemployment, %	8.77	9.00	9.33
	At least one parent unemployed, %	5.69	7.01	4.54
	Mean life satisfaction	7.58	7.48	7.53
	Sample size, n	8004	6984	5938
Germany	National unemployment, %	8.04	10.76	
	At least one parent unemployed, %	3.95	7.41	
	Mean life satisfaction	7.53	7.33	
	Sample size, n	5389	6951	
Greece	National unemployment, %		9.68	10.64
	At least one parent unemployed, %		3.49	6.08
	Mean life satisfaction		8.02	7.85
	Sample size, n		3617	4743
Hungary	National unemployment, %	5.52	7.37	10.78
	At least one parent unemployed, %	5.15	8.41	10.87
	Mean life satisfaction	7.55	7.26	7.41
	Sample size, n	3920	3369	4653
Iceland	National unemployment, %		2.69	7.37
	At least one parent unemployed, %		0.58	3.43
	Mean life satisfaction		7.80	7.97
	Sample size, n		9168	10,671
Ireland	National unemployment, %	4.18	4.46	13.03
	At least one parent unemployed, %	2.28	3.26	8.26
	Mean life satisfaction	7.61	7.72	7.60
	Sample size, n	2804	4616	4292
Italy	National unemployment, %	8.84	7.42	8.20
	At least one parent unemployed, %	3.54	3.67	5.10
	Mean life satisfaction	7.43	7.52	7.53
	Sample size, n	4281	3869	4734
Latvia	National unemployment, %	13.23	8.71	19.93
	At least one parent unemployed, %	7.33	3.85	10.89
	Mean life satisfaction	7.01	6.94	7.33
	Sample size, n	3144	4083	4016
Luxembourg	National unemployment, %		4.57	4.83
	At least one parent unemployed, %		2.67	3.37
	Mean life satisfaction		7.49	7.65
	Sample size, n		4083	3654
Netherlands	National unemployment, %	3.21	5.60	4.83
	At least one parent unemployed, %	2.78	4.00	3.49
	Mean life satisfaction	8.14	7.88	7.99
	Sample size, n	4050	4087	4215
Norway	National unemployment, %	3.61	4.22	3.37
	At least one parent unemployed, %	2.67	3.15	2.28
	Mean life satisfaction	7.45	7.87	7.78
	Sample size, n	4711	4526	4173
Poland	National unemployment, %	19.08	16.52	8.95
	At least one parent unemployed, %	16.27	10.51	7.26
	Mean life satisfaction	7.35	7.30	7.22
	Sample size, n	6045	5323	4057
Portugal	National unemployment, %	5.30	8.86	11.37
	At least one parent unemployed, %	4.16	6.07	9.31
	Mean life satisfaction	7.40	7.39	7.49
	Sample size, n	2827	3823	3881
Slovakia	National unemployment, %		15.25	13.79
	At least one parent unemployed, %		4.86	7.44
	Mean life satisfaction		7.80	7.45
	Sample size, n		3616	4866
Slovenia	National unemployment, %	6.37	6.58	6.62
	At least one parent unemployed, %	6.71	5.71	5.71
	Mean life satisfaction	7.66	7.54	7.67
	Sample size, n	3789	4946	5273
Spain	National unemployment, %	10.82	8.70	18.85
	At least one parent unemployed, %	3.41	3.95	9.91
	Mean life satisfaction	7.68	8.05	7.96
	Sample size, n	5616	8551	4750
Sweden	National unemployment, %	5.86	7.64	8.80
	At least one parent unemployed, %	4.35	5.75	5.74
	Mean life satisfaction	7.59	7.81	7.72
	Sample size, n	3523	4299	6333
Turkey	National unemployment, %		9.15	11.85
	At least one parent unemployed, %		4.66	6.77
	Mean life satisfaction		6.75	6.65
	Sample size, n		5135	4917
USA	National unemployment, %	5.34	4.86	9.77
	At least one parent unemployed, %	7.93	5.18	7.74
	Mean life satisfaction	7.47	7.44	7.52
	Sample size, n	4478	3654	5844
United Kingdom	National unemployment, %	5.07	5.03	7.81
	At least one parent unemployed, %	4.40	3.63	6.28
	Mean life satisfaction	7.43	7.38	7.50
	Sample size, n	13,105	14,304	14,497

### Ethics

This study uses secondary, publicly available data. The use of secondary data for this project was ethically reviewed by *The Ethical Review Board in Umeå*, Umeå University, Sweden (reference nr: 2017/282–31).

The original data collection underwent ethical review and gained approval in each separate country according to national regulations, and sometimes in schools [22, 24, 25]. Student participation in HBSC surveys was voluntary and active or passive consent was sought from school administrators, parents and children as per national human participant requirements. Responding children and their parents were given standardized information about the survey and that it was voluntary. The exact procedure for obtaining informed consent differed somewhat between countries, based on differing requests from national ethical committees. More information can be found in the international report from 2009/10 [22] and at the documentation section of the website for the HBSC Data Management Centre [24, 25].

The survey was anonymous and the data files that are provided by HBSC on their data bank contain no personal identifiers and no school identifiers; access to data files are given only after registration on the website, and we have kept data files secure on one computer.

### Measures

#### Life satisfaction

Life satisfaction, the outcome measure, was captured by the question: *Here is a picture of a ladder—the top of the ladder 10 is the best possible life for you and the bottom is the worst possible life—in general where on the ladder do you feel you stand at the moment?* This is an adapted version of the Cantril ladder, which has shown acceptable reliability and convergent validity among 11–15 year olds in Scotland [26].

The variable was left-skewed and somewhat truncated at the highest value, but more so among the 11-year olds (skewness − 1.21) than among the 13-year olds (skewness − 1,00) and 15-years old (skewness − 0.89). See Additional file 1 for histograms.

#### National unemployment

International datasets for unemployment are based on combinations of data from national employment office records, censuses and labour force surveys. The variable used here was “Harmonised Unemployment Rate” (HUR), computed by the OECD with the specific intent to be comparable internationally and across time, and available from the OECD open data repository [27]. The HUR defines unemployment as the percentage of the workforce “of working age who are without work, are available for work, and have taken specific steps to find work.” Using monthly averages as reported by OECD, the average unemployment rate during the 12 months from May–April during each survey school-year was computed.

Gross Domestic Product (GDP) per capita was considered for inclusion but was not used due to its high correlation with national unemployment (*r* = − 0.755 for the natural log of GDP per capita in purchasing power parities). This is expected given Okun’s law, which predicts a close relationship between unemployment and national production [28]. Gross National Income (GNI) per capita was somewhat less correlated with unemployment (r = − 0.517 for the natural log of GNI per capita) and was thus used for sensitivity analysis (not in table). Both GNI and GDP were extracted from the World Bank open data repository.

To distinguish between-country cross-sectional effects from within-country longitudinal effects, we followed a procedure recommended by Fairbrother [20], and used previously on similar research questions [8, 29]: national unemployment was separated into the country mean level (same for all years) and a country-mean-centered variable (different at each survey wave), resulting in two orthogonal, uncorrelated variables. For each country, the country-mean was computed as the average across all contiguous years from 2 years before the first survey-wave up to and including the year of the last survey-wave. The group-mean-centered unemployment used the measure for the relevant survey-year minus the country mean. This procedure also enables including several survey waves without the risk of bias due to correlation between a time-varying national variable and the country-level random intercept (also called endogeneity) [30], i.e. that the average level of unemployment could correlate with the rate of change in unemployment.

“Survey wave” as a confounder was used as a categorical variable, since the association with time was expected to be non-linear.

#### Individual independent variables

Parental unemployment in this study is defined as: parents being without job and looking for a job. Respondents first answered a question for each parent separately on whether he or she was working or not. If the answer was “yes”, there were follow-up questions on the place and type of job (not used here), and if the answer was “no”, there was a follow-up question on why he or she did not work, with the options “sick, retired or studying”, “looking for a job”, “is a stay-at home mom/dad” or “I don’t know”.

We recoded this into one combined variable with the categories unemployment (=“looking for a job”) of only mother, only father or both parents. The parents who were in the opposite category, i.e. “not unemployed” could thus be parents who were working (which was the majority, see Additional file 2), but they could also be studying, sick, being a stay-at-home parent, unknown or absent from the adolescent’s life completely. More adolescents reported an absent father (11,611) than an absent mother (1847). When *both* parents were absent or unknown, this was coded as missing (excluded from analysis, *n* = 9804). Additional file 2 contains information on the exact coding of this variable, and how many respondents reported the various combinations of parental situations.

Individual potential confounders were self-reported sex, age group and family situation, i.e. whether the mother and father lived in the same home or not, recoded into “separated parents”. Note that “separated parents” differs from having a missing parent, in that respondents who still had contact with both separated parents would report the employment situation for both.

### Analyses

Four-level mixed models linear regression analyses were used [20, 31, 32], with individuals nested in schools, country-years, and countries [20]. Since individual data is cross-sectional, each individual belongs to only one country-year.

As recommended by Fairbrother [20], and since used by Buffel et al. [8] and Kim & Hagquist [29] on similar research questions as ours, national unemployment was disaggregated into country mean unemployment and mean-centered yearly unemployment, and thus the risk of endogeneity was avoided. The country mean assesses the difference between countries, while the mean-centered variable assesses differences within countries over time.

To account for the respondents being clustered in schools, “school” was included as a level in the analysis. A grouping variable for schools (anonymized) was available in the dataset. For Germany in 2002 and Slovakia in 2006, the school variable was missing, and “school” was treated as only one group within those country-years. Though sample weights were available to compensate for the clustered sampling, these were not used since we did multilevel analysis (with schools as one level) to compensate for the clustered sample, and the weights were intended for single-level analyses (not divided by level), and thus not suited for this analysis.

The role of parental unemployment for the association between national unemployment and adolescent life satisfaction was analysed in three steps: First through bivariate correlations between national unemployment, aggregated percentage maternal/paternal unemployment, individual maternal/paternal unemployment, and average adolescent life satisfaction. Individual-level variables were aggregated to be measured at the same level – means and percentages for country level, deviations from mean at country-year level (Table 2), − while correlation with individual maternal/paternal unemployment was calculated using point-biserial correlations, since those variables are binary. Scatter plots for aggregated gender-specific parental and national unemployment are available in Additional file 3.

**Table 2:** Bivariate correlations between national unemployment rates^a^, aggregated percentage HBSC^b^ respondents with unemployed parents, and aggregated average life satisfaction of HBSC respondents (*n* = 386,402), in 27 countries across 3 waves (2002, 2005, 2009)

Correlations for all countries (*n* = 27 countries averaged across 2–3 waves per country) The survey variables (life satisfaction and parental unemployment) are aggregated across all waves for each country. National unemployment is the average national unemployment for all years from 2 years before first wave up until and including the year of the latest survey.		
	National unemployment^a^	Life satisfaction, average
% unemployed mothers	0.6639 (*p* < 0.001)	−0.2307 (0.247)
% unemployed fathers	0.6276 (*p* < 0.001)	−0.6120 (*p* < 0.001)
Life satisfaction, average	−0.3930 (*p* = 0.043)	
Correlations for all country-years (*n* = 74 country years) All variables are mean centered around the country mean. Survey variables (life satisfaction and parental unemployment) are aggregated by each country-wave, then mean-centered around the average for all waves per country.		
	National unemployment^a^	Life satisfaction, average
% unemployed mothers	0.7639 (*p* < 0.001)	0.0461 (*p* = 0.697)
% unemployed fathers	0.9094 (*p* < 0.001)	0.0335 (*p* = 0.777)
Life satisfaction, average	0.1496 (*p* = 0.203)	

^a^National unemployment is the variable Harmonized Unemployment Rate (HUR) from the OECD

^b^*HBSC* The survey Health Behaviour in School-aged Children

Second, by including parental unemployment as a control variable in the regression analysis of national unemployment in relation to life satisfaction (Table 3, Model 2). Third, by including interaction terms between parental unemployment and national unemployment, mean across all years, in relation to life satisfaction (Table 3, Model 3).

**Table 3:** Linear mixed models regression of national and parental unemployment in relation to life satisfaction^a^ of 11, 13 and 15-year olds (*n* = 386,402) in 27 countries across 3 waves (2002, 2005, 2009)^b^

		n (%)	Empty model	Model 1	*p*-value	Model 2	*p*-value	Model 3	*p*-value
Constant			7.58	9.13	< 0.001	9.12	< 0.001	9.12	< 0.001
Fixed effects									
Country overall unemployment				−0.04	0.011	−0.04	0.014	− 0.04	0.016
Year-specific unemployment				0.01	0.213	0.01	0.127	0.01	0.119
Sex (girl)		197,892 (51.2)		−0.18	< 0.001	−0.18	< 0.001	− 0.18	< 0.001
Age	11 years	127,083 (32.9)		1		1		1	
	13 years	132,581 (34.3)		−0.45	< 0.001	− 0.45	< 0.001	−0.45	< 0.001
	15 years	126,738 (32.8)		−0.77	< 0.001	−0.77	< 0.001	− 0.77	< 0.001
Wave	2001/02	107,786 (27.9)		1		1		1.00	
	2005/06	142,024 (36.8)		0.05	0.212	0.05	0.229	0.05	0.234
	2009/10	136,592 (35.4)		0.04	0.327	0.04	0.337	0.04	0.350
Family situation	Parents separated	97,219 (25.1)				−0.46	< 0.001	−0.46	< 0.001
Unemployed parents	None	365,848 (94.7)				1		1	
	Father	7156 (1.9)				−0.38	< 0.001	− 0.17	0.007
	Mother	12,294 (3.2)				−0.21	< 0.001	−0.25	< 0.001
	Both parents	1104 (0.3)				−0.49	< 0.001	−0.08	0.636
Interactions									
Country unemployment * Father unemployed								−0.02	0.001
Country unemployment * Mother unemployed								0.004	0.547
Country unemployment * Both parents unemployed								−0.04	0.005
Random effects									
Country variance (% change from previous model)^d^			0.076	0.049 (−34.9%)		0.049 (−1.1%)		0.049 (−0.2%)	
Country-year variance (% change from previous model)^d^			0.015	0.016 (8.7%)		0.016 (−0.8%)		0.016 (−0.4%)	
School variance (% change from previous model)^d^			0.164	0.068 (−58.6%)		0.067 (−0.5%)		0.067 (0%)	
Individual variance (% change from previous model)^d^			3.296	3.217(−2.4%)		3.213(−0.1%)		3.213 (0%)	
Log likelihood			− 784,253.4	− 779,415.8		− 776,821.08		−776,811.3	
Likelihood ratio test^c^			*p* < 0.001	*p* < 0.001		*p* < 0.001		*p* < 0.001	

^a^Life satisfaction is self-reported on a scale of 1–10, with higher values representing better life satisfaction

^b^27 countries (n: average 14,311.2, range 7737-41,906); 74 country waves (n: average 5221.6, range 2804-14,497); 14,344 schools/classes (n: average 26.9, range 1–5389)

^c^Likelihood ratio test compares each nested model to the previous model to see if it is a significantly better fit. The empty model is compared to a single-level regression

^d^The percentage change is obtained by dividing the difference in variance between a preceding model and a nested model by the variance in the preceding model

Analyses were performed with MLwiN through STATA version 12.1, using the command runmlwin [33].

Mixed effects models produce not only coefficients for associations (termed *fixed effects)*, but also estimates of how much of the variation is attributable to each level of analysis (termed *random effects*). As suggested by Merlo et al. [34] we present the variance by level, the percentage of variance by level (by dividing the variance at each level by the total variance) and the percentage of level-specific variance explained by including additional variables in nested models (Table 3). Thus, we also start with an empty multilevel model, which shows how much of the variation in outcome is attributable to different levels before including any independent variables.

Model fit was compared using log likelihood ratio test, which shows if a nested model is a significantly better fit to the data than the previous model, by computing -2ln(likelihood of first model/likelihood of nested model), using STATA:s command lrtest. The empty model was compared to a single-level model (linear regression).

## Results

### Descriptive statistics and bivariate correlations

The respondents were evenly distributed between survey years, age groups and boys/girls. A majority did not have any unemployed parents (94.7%), while 1.9% had only an unemployed father, 3.2% only an unemployed mother, and 0.3% had parents that were both unemployed. 25% had separated parents.

**Fig. 1:**
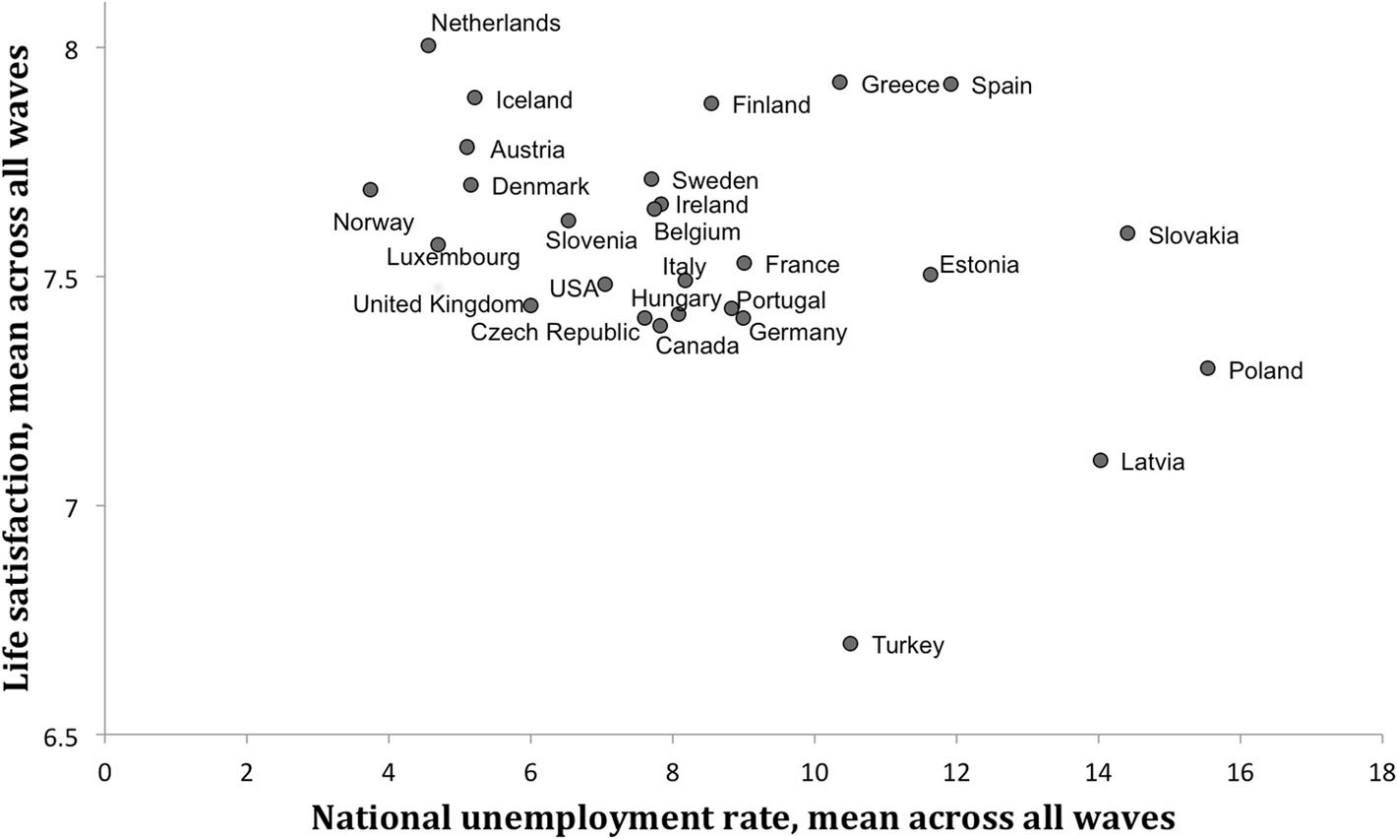
Life satisfaction versus national unemployment rates, by country, averaged across 3 waves (2002, 2005, 2009). ^1^ Life satisfaction from the HBSC survey (1–10) is averaged within each country across all three survey waves. ^2^ Harmonized Unemployment Rate (HUR) from the OECD is averaged within each country across the study period for that country

**Fig. 2:**
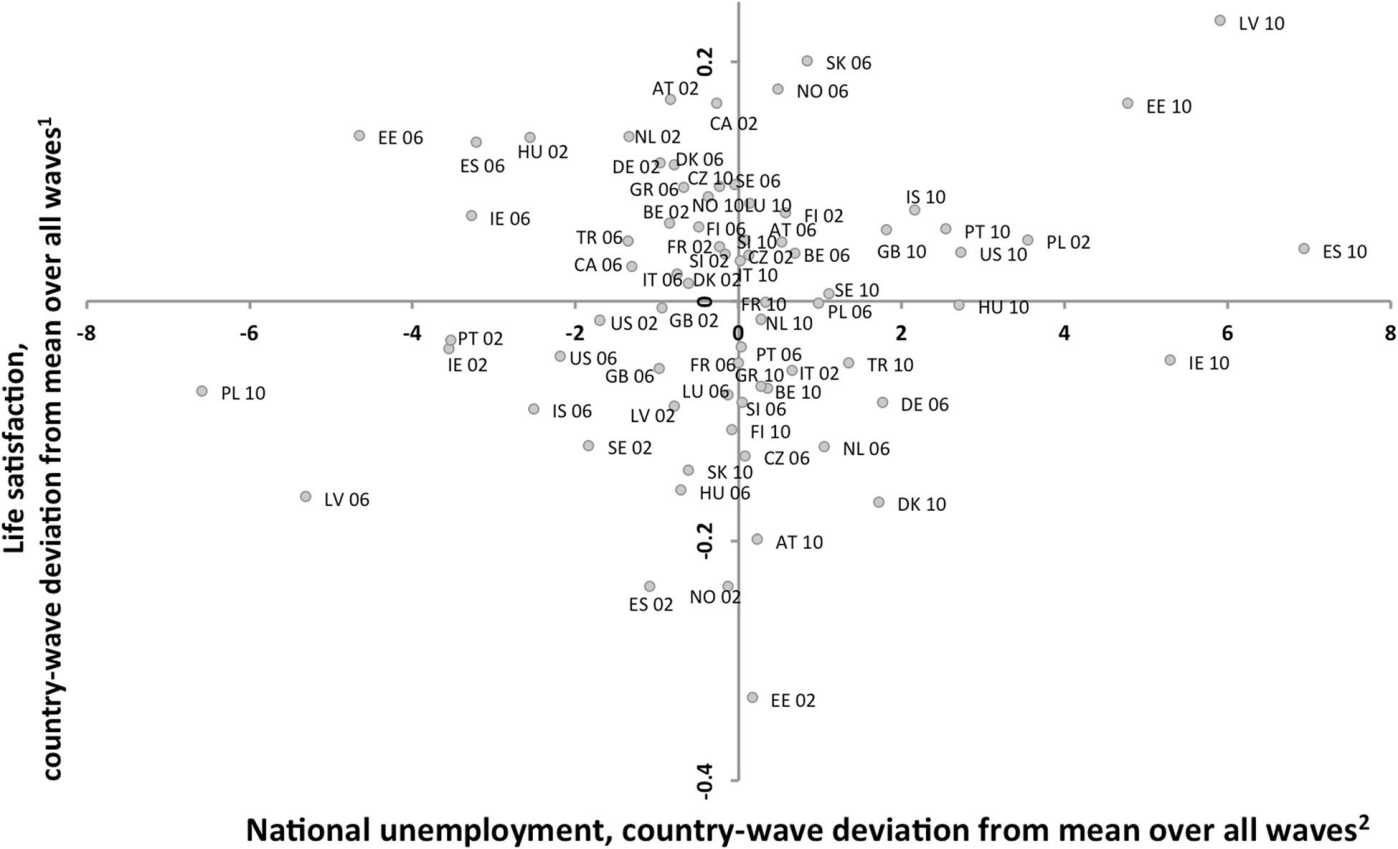
Life satisfaction versus national unemployment, by country-year. Each country-wave is mean-centered in relation to the mean for all waves, in 27 countries across 3 waves (2002, 2005, 2009). AT = Austria, BE = Belgium, CA = Canada, CZ = Czech Republic, DE = Germany, D = Denmark, EE = Estonia, ES = Spain, FI = Finland, FR = France, GB = United Kingdom, GR = Greece, HU = Hungary, IS = Iceland, IE = Ireland, IT = Italy, LU = Luxembourg, LV = Latvia, NL = Netherlands, NO = Norway, PL = Poland, PT = Portugal, SE = Sweden, SI = Slovenia, SK = Slovakia, TR = Turkey, US = USA

National unemployment rates varied widely across countries and years, as shown in Table 1 and Figs. 1 and 2, from Norway with consistently low unemployment (on average 3.74%, slightly higher in 2006), to Poland with high but decreasing unemployment (15.54% on average, decreasing from 19.08% in 2002 to 8.95% in 2010). The lowest point estimate was 2.69% in Iceland in 2006 and the highest 19.93% in Latvia in 2010.

Correlation between national unemployment and country-level percentage of parental unemployment was high, on country level 0.66 for aggregated maternal unemployment and 0.63 for aggregated paternal unemployment, on country-year level 0.76 for maternal unemployment and 0.91 for paternal unemployment (Table 2). These strong correlations were only with *aggregated* parental unemployment though. Point-biserial correlations between national unemployment and individual parental unemployment were significant but small, country level national unemployment was correlated 0.0525 (*p* < .001) with mothers’ and 0.0457 (*p* < .001) with fathers’ unemployment, mean-centered yearly national unemployment correlated 0.0387 (*p* < .001) with mother’s and 0.0606 (*p* < .001) with father’s unemployment (data not in table). National unemployment as well as percentage of parental unemployment were significantly and negatively correlated with life satisfaction across countries for all years, but not in change across years; see also Figs. 1 and 2.

### Mixed models

#### Fixed effects

Country-level unemployment (mean across years) was associated with lower adolescent life satisfaction, an association that remained significant after controlling for parental unemployment (Table 3). Country-year unemployment (mean-centered) was not related to adolescent life satisfaction.

Interaction analysis between national unemployment (mean across all years) and individual parental unemployment showed that the association between national unemployment and life satisfaction was stronger among adolescents with an unemployed father or both parents unemployed, compared to adolescents with no unemployed parents. For adolescents with an unemployed mother the association between national unemployment and life satisfaction was virtually the same as among respondents with no unemployed parents.

Having unemployed parents (father, mother, or both parents) was in itself associated with lower life satisfaction. When adding the interaction term, the association of mothers’ unemployment to life satisfaction remained strong, while the association of fathers’ or both parents’ unemployment decreased (becoming non-significant for the latter).

Being a girl, older age and having separated parents was associated with lower life satisfaction.

Sensitivity analyses showed that results remained significant, though with smaller coefficients, after controlling for log-transformed GNI (not in table).

#### Random effects

Table 3 also shows that the total variation in life satisfaction before introducing explanatory variables (i.e. in the empty model) was 3.551, of which 0.076 (2.14% of the total variation) was attributable to variation between countries, 0.015 (0.42%) to variation over years within countries, 0.164 (4.62%) to variation between schools, and 3.296 (92.82%) to variation between individuals. Controlling for national unemployment, sex, age, separated parents and survey-wave (Model 1), explained over a third of the country-level variation, almost 60% of the school level variation, and 2.4% of the individual variation. Adding parental unemployment (Model 2) and the interaction (Model 3) explained little of the remaining variation. Country-year variation remained stable across models.

## Discussion

Higher national unemployment was associated with lower adolescent life satisfaction, cross-sectionally across countries but not over time within countries. Notably, this applied whether the parents were unemployed or not, although stronger among adolescents with unemployed fathers or with both parents unemployed. There was no *additional* effect of national unemployment for those only having an unemployed mother, though mothers’ unemployment was independently related to lower life satisfaction.

Adolescent life satisfaction is an important indicator not only of general wellbeing, but also of mental health and the absence of mental ill health [13]. Some of the many positive effects of high life satisfaction in this age group is enhanced social and academic functioning, better academic achievements, and resilience against stressful life situations [35–38].

Some have argued that the effects of macroeconomics on children and adolescents are wholly mediated by the situation within the immediate family [9, 10, 39], which the current study contradicts for 11–15-year olds. There could of course be other family-level mediators explaining the effect of national level unemployment on adolescent life satisfaction, such as parents’ stress due to an increasingly insecure labour market, or unemployment of other family members.

National unemployment has to our knowledge not previously been analysed in relation to adolescent life satisfaction. There is, however, research relating adolescent *mental and somatic health complaints* to various measures of national unemployment [5, 40, 41]. That research shows that in adolescence, such complaints (sadness, anxiety, irritability, sleeping problems, headache, stomachache, backache and dizziness) are mainly associated with national levels of *youth* unemployment [5, 40], or other labour market exclusion of youth [41] but not with adult unemployment rates. Further research is needed to understand if these differences of results are due to the health outcome studied.

The relationship between unemployment in relation to health or wellbeing could differ between countries [17, 19, 42]. There could be many factors explaining such differing associations, for example the level of unemployment benefits, which can be protective for the health of persons becoming unemployed [19]. One important direction for future research is to what extent unemployment benefits and other social security nets can alleviate effects of national and parental unemployment on life satisfaction and mental and physical health of adolescents.

Studies indicate that for adults, regional and local unemployment rates within countries could also matter for health and well-being [43], and for the relationship between own unemployment and health [44, 45]. Since our samples are nationally representative, the regional or local variation should not affect our conclusions in any major way. Two different studies in Sweden found no association between local unemployment rates and adolescent psychosomatic symptoms [29].

That parental unemployment is related to lower adolescent life satisfaction has been shown in earlier research [5, 40], but the interaction with national unemployment has not been studied. The finding that national unemployment had a stronger association to life satisfaction among adolescents with fathers or both parents unemployed could be because high national unemployment implies a lower chance that their parent(s) will regain employment soon. Among adults, including young adults, being unemployed during a time of high general unemployment levels compared to periods with low unemployment levels can in some instances be protective (maybe due to lower stigma, or differing composition of the unemployed population), in other instances negative [12, 42, 44, 46].

Regarding individual or family-level unemployment, it cannot be exluded that health selection is partly or mainly responsible for the association between being unemployed and self-reported health and well-being of the unemployed person, i.e. that persons with lower health status are more likely to become unemployed [16, 17, 47]. For adolescents, it is unlikely that their life satisfaction per se would affect their parents’ employment status except in extreme cases; but low adolescent life satisfaction could be a reflection of pre-existing parental illness or psychosocial problems in the family, which in turn could have led to unemployment of the parent(s). *National* adult unemployment, which is the main variable of interest here, is even less likely to be affected by adolescent life satisfaction.

Health selection might be less prominent when unemployment rates rise steeply, due to changes in the drivers of job-loss. One European study shows that in countries that combined high national unemployment and a recent sharp increase in the unemployment rate, the unemployed population on average had fewer limiting longstanding illnesses compared to in countries with low and/or or stable unemployment, which was interpreted to be a compositional effect – when the labour market is insecure, everyone is at higher risk of losing their employment, even those in good health [42].

The unemployed parents are here contrasted not only with working parents but also with parents who were sick, retired, students or stay-at-home parents. The reason for this coding was that we assumed that unemployment carries its own stigma. However, having parents who are home due to illness could have an even heavier effect on adolescent life satisfaction; something to explore in future research. The effect of having a stay-at-home parent could differ depending on gender norms of the country; also, in a country where stay-at-home moms are a norm, women who lose their job might go back to considering themselves a stay-at-home parent even when they would prefer to work.

Notably, we found no added effect of national unemployment on life satisfaction when the adolescent exclusively had an unemployed mother. This should thus be considered in relation to gendered norms for work and stay-at-home parents [48]. In settings where stay-at-home moms are the norm, adolescents might consider an unemployed mother equivalent to a stay-at-home mom, and national unemployment may be considered irrelevant in relation to one’s own mother being at home. However, there was a negative assosiation with life satisfaction of having an unemployed mother, and of national unemployment, but independently. The gender aspect needs to be explored further, in relation to gender-specific national unemployment, family composition, stay-at-home moms, and gender norms of the country.

The lack of associations across time is in accordance with a previous study on adolescents using HBSC data. This study found associations between health complaints and *levels* of youth unemployment but not *changes* therein [5]. It is also in accordance with a study on adults’ mental health and healthcare, using the same analytic method as here and also using the years 2002, 2005 and 2010 (with Eurobarometer data), where the country mean unemployment was related to lower self-reported mental health of both employed and unemployed men and employed women; but the mean-centered unemployment variable was not related to mental health [8]. The statistical power to detect associations across time depends on the number of waves included and the size of the variation over time [20]. In our case, we had only three waves, and the variation in life satisfaction over years within countries was relatively small, both of which might explain the lack of associations of unemployment with life satisfaction across time.

However, it is possible that the association we have detected is a proxy for other, systemic between-country differences. One direction for future research is to explore this over longer time. It is also possible that associations might be non-linear, for instance due to threshold effects, i.e. adolescents may not be aware of changes in national unemployment rates until those rates reach a crisis level.

### Strengths and limitations

One strength of our study is that the HBSC has a large sample, representing adolescents in numerous countries and over several years with different levels of unemployment. The statistical method used here allows for the inclusion of more than one survey wave, resulting both in a larger sample with greater variation to be analysed, and the possibility to analyse time and geography separately [20]. The method has only occasionally been used in public health, but has recently gained interest in social and geographical science [8, 20].

The validity of our results is supported by the use of a standardised measure of national unemployment. Unemployment statistics can differ depending on national definitions; but the variable used here has been adjusted by the OECD to be internationally comparable. Though this variable was available for fewer countries, we preferred that over using a less comparable variable.

It should be mentioned that several macroeconomic conditions have such a close relationship with unemployment that they are difficult to disentangle from each other. Although our results did hold when controlling for GNI per capita, we cannot exclude that national unemployment may function as a proxy for other national macroeconomic characteristics.

Self-reported data can be both a strength and a weakness. For example, adolescents may be unaware whether their non-working parents are actively looking for work. We assume that this would underestimate rather than overestimate parental unemployment: when adolescents report that the parent is looking for a job, it is probable that this has been a topic of conversation at home; but if the parent is not discussing their job search at home, the adolescent might instead erroneously report them as sick or a stay-at-home parent, in which case we would not code them as unemployed. The strong correlation between aggregated parental unemployment and national unemployment supports the validity of the self-reported parental unemployment.

Exposure and outcome variables are from independent sources, minimizing the risk of shared method variance (except that both parental unemployment and life satisfaction are self-reported).

The questionnaires have been carefully translated to national languages in such ways as to ensure comparability. Even so, certain variables, especially life satisfaction, could be perceived differently in different countries and languages. However, self-reported life satisfaction is a well-established measure, used a lot in previous research on adolescents [13, 14, 35]. We used it as a linear variable, to enable quantifying variation on different levels. The variable is discrete and bounded, not continuous and unbounded. However, linear regression is quite robust to minor violations of assumptions, and life satisfaction in the HBSC survey has been used as a linear variable previously [15, 49].

The survey is cross-sectional and thus causality on individual level cannot be inferred.

## Conclusions

Living in a country with higher national unemployment across the years seems to be associated with lower adolescent life satisfaction, and this seems to apply whether their own parents are unemployed or not, although stronger among adolescents where the father or both parents are unemployed. However, variation in unemployment over the years did not show an association with adolescent life satisfaction.

Adolescent life satisfaction is vital for for adolescents’ mental health, quality of life and academic performance; all of which are burning issues for national policy makers. Findings of potential independent effects of national macroeconomic factors on adolescent life satisfaction open up new avenues for national policy, but this needs to be explored further in future research.

The conclusions here highlight the question of the societal costs of unemployment, above and beyond immediate economic costs such as lowered tax revenue and expenses for unemployment benefits and other social welfare. If, as our results indicate, national circumstances might be related to adolescent current life satisfaction, this can be considered not only a social cost in the present but also a potential social and economic cost for the future, if adolescents’ current life satisfaction has an impact on their education performance, life choices, or future mental health.

Other impacts of economic cycles are important to consider. One study shows that an increase in adolescents’ psychosomatic symptoms in a region of Sweden was largely explained by an increase in their worries about family finances during the Swedish economic crisis of the early 1990’s (but not at later time points) [29].

Another aspect to consider for policy makers is the importance of the unemployment of both men and women. Often, the employment status of fathers is given prominence, especially in countries with a sole-breadwinner model. Based on our results, it is important to note that not only paternal but also maternal unemployment shows a negative association with adolescent life satisfaction. In our study, though the negative effect of fathers’ unemployment was somewhat stronger, the unemployment of mothers was higher than that of fathers, meaning it affected more adolescents.

## Supplementary information

**Additional file 1: Figure S1.** Histogram of self-reported life satisfaction, by sex and age group, *n* = 386,402.

**Additional file 2: Table S1.** Content (coding) of the variable for “Unemployed parents”, n = 386,402.

**Additional file 3: Figure S2.** Gender specific scatter-plot of general versus parental unemployment by country.

## Data Availability

The HBSC data for the survey waves used here is publicly available; no permission is needed, but one needs to register a user profile with a functioning email address in order to access the data. (For survey waves before 2002, special permission is needed, but those waves were not used here.) By registering a user profile, one is assumed to agree to the license agreement, as written here: https://www.uib.no/en/hbscdata/94218/license-agreement-use-data-hbsc-data-bank The data is accessed through the HBSC website from www.hbsc.org, which redirects to the data bank website https://www.uib.no/en/hbscdata which in turn links to the database interface http://hbsc-nesstar.nsd.no/webview/ where the data can be accessed after registering a user profile. The national Harmonized Unemployment Rate is publicly and openly available from data.oecd.org or more specifically https://data.oecd.org/unemp/harmonised-unemployment-rate-hur.htm No registration is needed. In this study, we used the monthly data, and recalculated it to yearly data to roughly match the school year (we calculated mean unemployment for may-april). The Gross National Income by country (used for sensitivity analyses) is publicly and openly available from the same source.

## Abbreviations

GDP: Gross Domestic Product
GNI: Gross National Income
HBSC: Health Behaviour in School-aged Children (a survey)
OECD: Organisation for Economic Co-operation and Development (a group of currently 36 member countries)
WHO: World Health Organization
