# Supplementary figures and images for: The interplay between national and parental unemployment in relation to adolescent life satisfaction in 27 countries: analyses of repeated cross-sectional school surveys
Source: BMC Public Health. 2019 Nov 28;19:1555. doi: 10.1186/s12889-019-7721-1 (PMC6882305; doi:10.1186/s12889-019-7721-1)

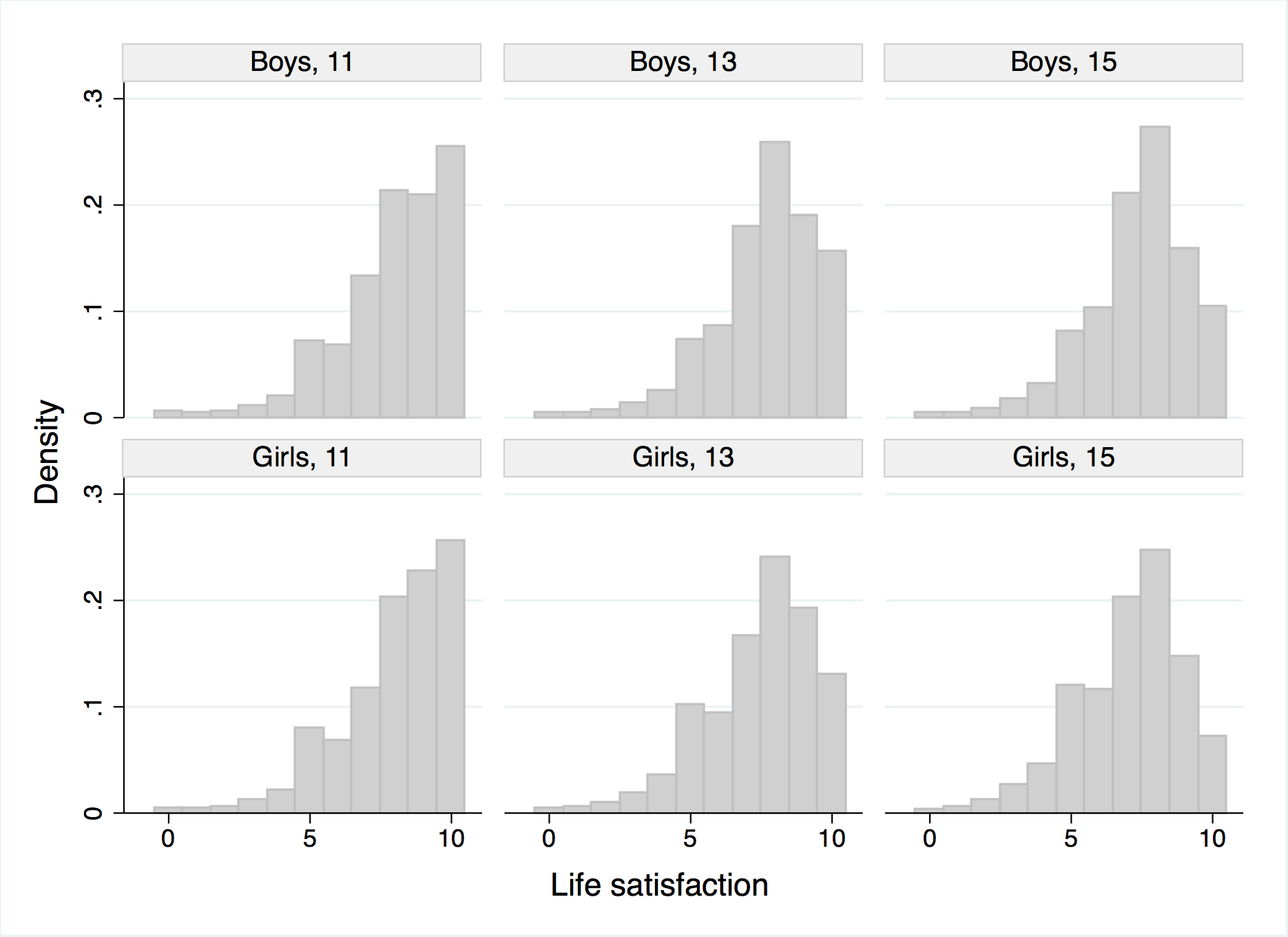

Supplement: Supplementary file 1 — Additional file 1: Figure S1. Histogram of self-reported life satisfaction, by sex and age group, n = 386,402. [file 12889_2019_7721_MOESM1_ESM.tif]

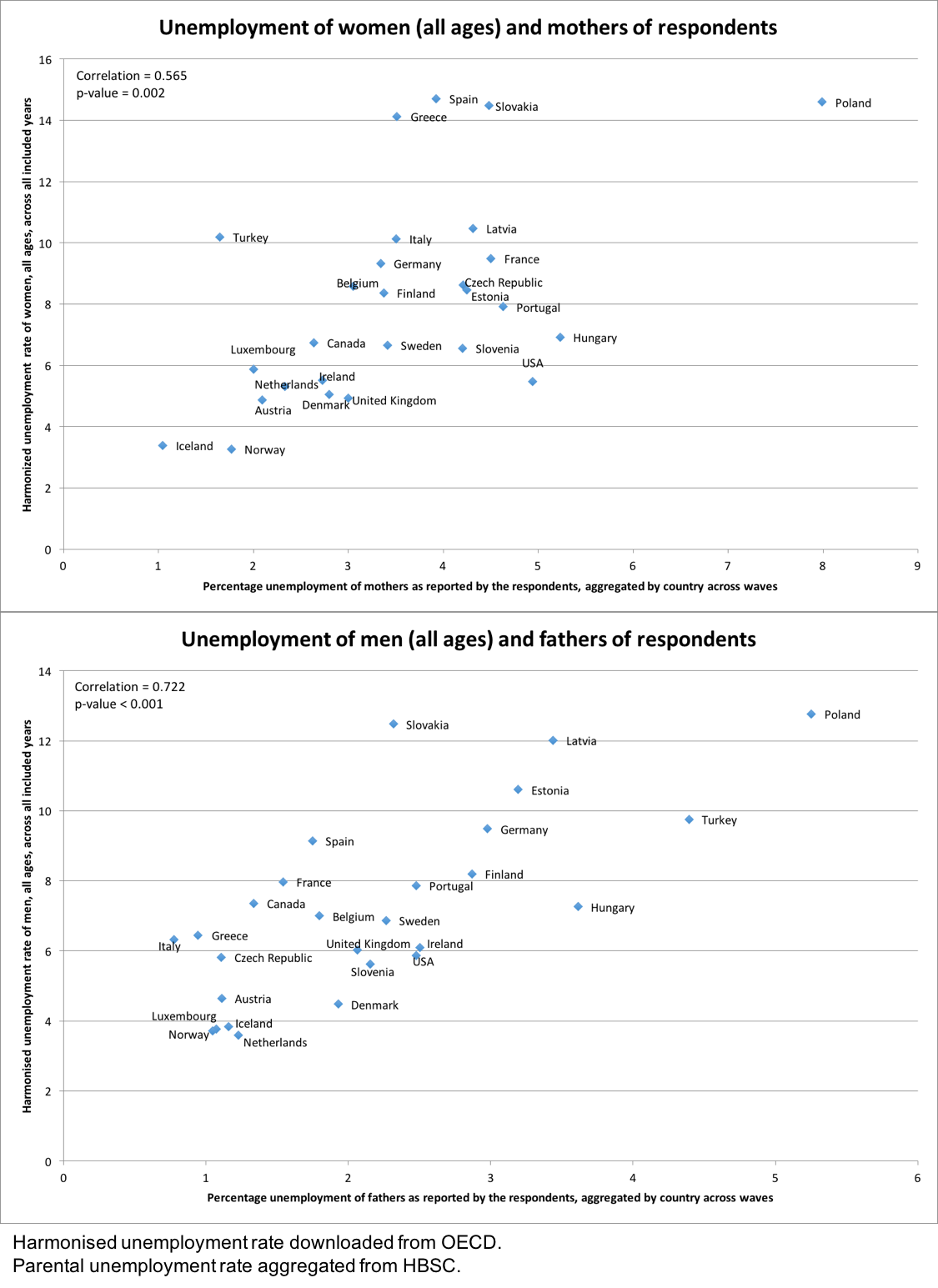

Supplement: Supplementary file 3 — Additional file 3: Figure S2. Gender specific scatter-plot of general versus parental unemployment by country. [file 12889_2019_7721_MOESM3_ESM.png]
