# Supplementary material for: The interplay between national and parental unemployment in relation to adolescent life satisfaction in 27 countries: analyses of repeated cross-sectional school surveys
Source: BMC Public Health. 2019 Nov 28;19:1555. doi: 10.1186/s12889-019-7721-1 (PMC6882305; doi:10.1186/s12889-019-7721-1)
Supplement: Supplementary file 2 — Additional file 2: Table S1. Content (coding) of the variable for “Unemployed parents”, n = 386,402. [file 12889_2019_7721_MOESM2_ESM.docx]

**Additional file1: Table S1. Content (coding) of the variable for “Unemployed parents”, n=386,402**

| **The category in our analyses:** | **The potential combinations of self-reported parental employment situations for both parents** |
| --- | --- |
| No parent unemployed  n= 365,848 | Neither of the parents is reported as “looking for a job” and at least one parent is non-missing, consisting of the following combinations: |
|  | - Both parents employed, n= 261,423 |
|  | - One parent employed, the other sick/retired/studying, n=12,903, or “home working”, n=41,579, or reported as “don’t know” or unclassifiable to coders, n=30,237, or absent from the respondent’s life, n=10,758 |
|  | -Both parents are either sick/retired/studying, or “home working”, n=3,884 |
|  | - One parent is sick, retired, studying, or “home working”, the other is reported as “don’t know” or was unclassifiable to coders, n=3,223 |
|  | - One parent sick, retired, studying, or “home working”, the other absent from the respondent’s life, n=1,841 |
| Father unemployed  n= 7,156 | Father reported as “looking for a job”, while mother reported as either employed (n=4,882), sick/retired/studying (n=341), “home working” (n=1,369), not classifiable (n=193), no contact (n=80) or don’t know (n=291). |
| Mother unemployed  n=12,294 | Mother reported as “looking for a job”, while father reported as either employed (n=10,093), sick/retired/studying (n=505), “home working” (n=94), not classifiable (n=324), no contact (n=779) or don’t know (n=499). |
| Both parents unemployed, n=1,104 | Both parents reported as “looking for a job”, n= 1,104 |
| Missing (excluded from analyses)  n=9,804^a^ | Both parents reported as “don’t know” (n=1,919), no contact (n=464), the non-classifiable response (5,919) or a combination of these three options (n=1,502) |

^a^ The N for missing refers only to those who are non-missing on all other variables in the survey.
